# HIF-1α inhibitor echinomycin reduces acute graft-versus-host disease and preserves graft-versus-leukemia effect

**DOI:** 10.1186/s12967-017-1132-9

**Published:** 2017-02-10

**Authors:** Yushi Yao, Lei Wang, Jihao Zhou, Xinyou Zhang

**Affiliations:** 1 MDCL-4084, Department of Pathology & Molecular Medicine, McMaster University, 1280 Main Street West, Hamilton, ON L8S4K1 Canada; 2 Department of Hematology, Shenzhen People’s Hospital, 1017 Dongmen North Road, Shenzhen, 518020 China; 3 Department of Hematology and Department of Clinical Nutrition, General Hospital of Chinese People’s Armed Police Forces, 69 Yong Ding Road, Beijing, 100039 China

**Keywords:** Acute graft-versus-host disease, Graft-versus-leukemia effect, Hypoxia-inducible factor 1 alpha, Echinomycin, Regulatory T cells, Th17 cells

## Abstract

**Background:**

Acute graft-versus-host disease (aGVHD) remains a major obstacle against favorable clinical outcomes following allogeneic hematopoietic stem cell transplantation (allo-HSCT). T helper cells including Th17 play key roles in aGVHD pathogenesis. Donor regulatory T cell (Tregs) adoptive therapy reduces aGVHD without weakening graft-versus-leukemia effect (GVL) in both mouse and human, although the purification and ex vivo expansion of Tregs in clinical scenarios remain costly and technically demanding. Hypoxia-inducible factor 1 alpha (HIF-1α) is a key molecule switch that attenuates Treg but promotes Th17 development. However, whether pharmacological inhibition of HIF-1α reduces aGVHD via increasing Treg development and diminishing Th17 responses remains unexplored.

**Methods:**

By using alloantigen-specific mixed lymphocyte culture and murine models of aGVHD and GVL, we evaluated the impacts of HIF-1α inhibition by echinomycin on the alloantigen-specific CD4 T cell responses ex vivo, as well as on aGVHD and GVL effect following allo-HSCT.

**Results:**

Ex vivo echinomycin treatment resulted in increased number of Tregs in the culture as well as reduced alloantigen-specific Th17 and Th1 responses. In vivo echinomycin treatment reduced GVHD scores and prolonged survival of mice following allo-HSCT, which is associated with increased number of donor Tregs and reduced number of Th17 and Th1 in lymphoid tissues. In murine model of leukemia, echinomycin treatment preserved GVL effect and prolonged leukemia free survival following allo-HSCT.

**Conclusions:**

Echinomycin treatment reduces aGVHD and preserves GVL effect via increasing donor Treg development and diminishing alloantigen-specific Th17 and Th1 responses following allo-HSCT, presumably via direct inhibition of HIF-1α that results in preferential Treg differentiation during alloantigen-specific CD4 T cell responses. These findings highlight pharmacological inhibition of HIF-1α as a promising strategy in GVHD prophylaxis.

**Electronic supplementary material:**

The online version of this article (doi:10.1186/s12967-017-1132-9) contains supplementary material, which is available to authorized users.

## Background

Allo-HSCT remains the only therapeutic strategy that has the potential to cure hematopoietic malignancies including leukemia [1, 2]. Therapeutic effects of allo-HSCT rely largely on GVL effect where donor T cells eradicate residual leukemia cells primarily in an alloantigen specific manner and thus prevent leukemia from relapse [3, 4]. However, aGVHD that is closely associated with GVL effect has been a huge challenge to favorable clinical outcomes following allo-HSCT [1, 4]. Similar to GVL effect, aGVHD results from donor T cell-mediated alloantigen-specific immune responses [5]. It has been shown that alloantigen-specific Th1 and Th17 cells contribute significantly to systemic inflammation and target tissue damage during aGVHD [6–8]. Based on these findings, T cell depletion from hematopoietic stem cell (HSC) graft represents an efficient strategy in aGVHD prophylaxis. Unfortunately, accumulating data have shown that such a pan-T cell targeting regimen results in weakened GVL effect as evidenced by the increased risk of graft failure, delayed immune reconstitution, and leukemia relapse [9–11]. For these reasons, improved prophylactic and/or therapeutic strategies are in urgent need to separate aGVHD and GVL.

In recent years, infusion of donor Tregs has been reported as a promising aGVHD prophylactic strategy in both mouse and human without weakening GVL effect [12–14]. In one of these studies, adoptive transfer of donor Tregs prevented GVHD in human subjects receiving allo-HSCT, with improved lymphoid reconstitution and immunity to opportunistic pathogens without weakening GVL effect [13]. In line with this, clinical studies revealed that higher number of Tregs in HSC graft are associated with reduced GVHD, and abundance of Foxp3 gene expression was significantly higher in non-GVHD than GVHD patients following allo-HSCT [15, 16]. These studies provided compelling evidence that prevention of GVHD without weakening GVL can be achieved via increasing the number of donor Tregs in HSC recipients. However, current clinical protocols for Treg purification and ex vivo expansion are costly and highly technically demanding [14]. Moreover, Tregs purified and/or expanded by using various protocols may represent different subtypes and thus have inconsistent functions in preventing GVHD and/or preserving GVL effect [17]. These obstacles remain challenges to the wide application of Treg infusion as a GVHD prophylactic regimen in clinical scenarios.

HIF-1α is a key metabolic sensor regulating the differentiation of CD4 T cells [18, 19]. It has been shown that HIF-1α inhibits Treg development via targeting Foxp3 for proteasomal degradation [18]. Concurrently HIF-1α enhances Th17 development via transcriptional activation of RORγt [18, 20]. As Tregs play regulatory roles not only in Th17 responses but also in other T helper cells including Th1 cells [21], HIF-1α serves as a molecular switch between Treg-mediated immune homeostasis and T helper cell-mediated immune responses and immunopathology. Indeed, mice with HIF-1α deficiency in CD4 T cells were resistant to experimental autoimmune encephalitis model, a Th17 dependent autoimmune disease. Such a phenotype is associated with increased Treg development and reduced Th17 responses [18]. Echinomycin, a small molecule HIF-1α inhibitor, reduces target molecule binding ability of HIF-1α [22, 23]. Inhibition of HIF-1α by echinomycin reduced Th17 development and persistence, which is consistent to that observed in HIF-1α deficient CD4 T cells [24]. However, it remains unexplored whether HIF-1α inhibition by echinomycin reduces aGVHD by promoting Tregs and diminishing Th17 development.

By using murine models of aGVHD and GVL, we show here that HIF-1α inhibition by echinomycin treatment significantly reduces aGVHD without weakening GVL, resulting in prolonged leukemia free survival. This phenotype is associated with increased donor Tregs and diminished Th17 and Th1 responses in lymphoid organs. Our study highlight the significance of HIF-1α in Treg/Th17 balance during aGVHD pathogenesis, and that pharmacological inhibition of HIF-1α represents a promising novel GVHD prophylactic strategy that is worthy of further investigation.

## Methods

### Mice

Wild-type C57BL/6J and Balb/c mice (female, 6–8 weeks of age) were purchased from the Joint Ventures Sipper BK Experimental Animal Co. (Shanghai, China). Mice were housed in specific pathogen-free conditions, and all experimental manipulations were conducted in accordance with the National Institute of Health Guide for the Care and Use of Laboratory Animals along with approval from the Scientific Investigation Board of the Shenzhen People’s Hospital.

### Leukemia cell line

A20 leukemia cells were a kind gift from Dr. Kai Sun from Henan Provincial People’s Hospital. Cells were cultured at 37 °C in a 5% CO_2_ incubator in RPMI 1640 culture media supplemented with 10% fetal bovine serum (HyClone Laboratories, South Logan, UT, USA), penicillin and streptomycin. To determine the apoptosis of A20 cells, cells were cultured in triplicates in 24-well plates for 24 h in the presence of echinomycin at indicated concentrations, followed by Annex V and PI staining. For colony forming assay, A20 cells were cultured in the presence of echinomycin at indicated concentrations for 24 h. Cells were then washed to remove echinomycin, and suspended in Methocult H4230 (STEMCELL Technologies, Vancouver, BC, Canada) at 1 × 10^3^ cells/mL, plated in 24-well plate in triplicates and cultured for 7–14 days at 37 °C in a 5% CO_2_ incubator. The colony forming units (CFU) per well was counted and representative pictures of cellular clusters were taken under a DMI6000 inverse microscope (Leica Biosystems, Heidelberg, Germany).

### Generation of bone marrow derived dendritic cells

Murine bone marrow derived dendritic cells (BMDCs) were generated as previously described [25, 26]. Briefly, murine bone marrow mononuclear cells were obtained from femur bones followed by red blood cell lysis. Bone marrow cells were then cultured for 7 days in RPMI 1640 culture media supplemented with 10% fetal bovine serum(HyClone Laboratories, South Logan, UT, USA), penicillin and streptomycin in the presence of 10 ng/mL murine GM-CSF and 1 ng/mL murine IL-4 (both from Peprotech, Rocky Hill, NJ, USA). On day 7, BMDCs were stimulated with 100 ng/mL LPS (Sigma-Aldrich, St. Louis, MO, USA) for 24 h to promote maturation. Mature BMDCs were then recovered and washed before coculture with T cells.

### Coculture of BMDCs with CD4 T cells

Murine CD4 T cell negative isolation kit (Miltenyi Biotech, Cambridge, MA, USA) was used to purify CD4 T cells from splenic single cell suspension following the manufacturer’s instructions. The purity of purified CD4 T cells was typically between 90 and 95%, as determined by flow cytometry analysis. After purification, 1 × 10^6^ CD4 T cells were cocultured with 1 × 10^5^ allogeneic BMDCs in triplicates in 24-well plates at 37 °C in a 5% CO_2_ incubator for up to 6 days. Echinomycin was supplemented once daily in the media at 1 nM of final concentration. Media supplemented with DMSO at the same volume of that in echinomycin dilution was used as media control.

### ELISA

At indicated time points of cell culture, supernatants were collected and preserved at −20 °C. Concentrations of cytokines in culture supernatants were determined by using mouse IL-2, IL-10, IL-17A and IFN-γ ELISA kits according to the manufacturer’s instructions (all from R&D Systems, Minneapolis, MN, USA).

### Flow cytometry staining and analysis

Unless otherwise specified, all reagents for flow cytometry were purchased from BD Biosciences (San Jose, CA, USA). For extracellular staining of CD4 T cells, anti-mouse H2Kb-FITC, anti-mouse CD3-V450, anti-mouse CD4-AF700, and anti-mouse CD25-APC antibodies were used. Fox extracellular staining of BMDCs, anti-mouse MHC-II-AF700 (eBioscience, San Diego, CA, USA), anti-mouse CD86-APC and anti-mouse CD40-FITC were used. For intracellular staining of Foxp3, anti-mouse Foxp3-PE antibody was used after fixation and permeabilization of cells with Foxp3/Transcription Factor Staining Buffer Set from eBioscience (San Diego, CA, USA). For intracellular staining of IL-17 and IFN-γ, cells were cultured in the presence of GolgiPlug (1:400) for 5 h before harvest. After fixation and permeabilization, cells were stained with anti-mouse IL-17A-PE or anti-mouse IFN-γ PE antibodies. For T cell proliferation analysis purified CD4 T cells were stained with CFSE (5 µM; eBioscience, San Diego, CA, USA) and cultured alone or with allogeneic BMDCs for 5 days. To determine the apoptosis of A20 cells, Annexin V Apoptosis Detection Kit (eBioscience, San Diego, CA, USA) was used. Cells were acquired on a LSR II flow cytometer (BD Biosciences, San Jose, CA, USA). Data were analyzed by using FlowJo software version 10 (TreeStar, Ashland, OR, USA), except for CFSE T cell proliferation analysis where FlowJo software version 7.6.1 (TreeStar, Ashland, OR, USA) was used.

### Murine aGVHD and GVL models

To generate murine aGVHD model, Balb/c mice were lethally irradiated with 850 cGy γ-ray. Immediately after irradiation, mice were infused with 5 × 10^6^ bone marrow cells supplemented with 1 × 10^6^ splenic T cells from C57BL/6 mice via the tail vein. For syngeneic HSCT control, identical numbers of bone marrow cells and splenic T cells from Balb/c mice were infused instead. EasySep Mouse T Cell Isolation Kit (STEMCELL Technologies, Vancouver, BC, Canada) was used to purify splenic T cells according to the manufacturer’s instructions. To generate murine GVL model, 1 × 10^6^ A20 cells were supplemented to bone marrow graft of either Balb/c (syngeneic) or C57BL/6 (allogeneic) mice as mentioned above, and infused into lethally irradiated Balb/c mice. Echinomycin was intraperitoneally injected at 5 µg/kg once every other day starting from day 1 after HSCT till moribund appearance or through the end of observation. PBS supplemented with DMSO at the same volume of that in echinomycin dilution was used as vehicle control. After HSCT, mice were monitored daily for physical appearance and body weight. GVHD score was given weekly based on weight loss, posture, activity, fur texture, and skin integrity as previously described [27]. In experiments shown in Fig. 5d, Balb/c mice were sublethally irradiated with 500 cGy γ-ray and infused with 1 × 10^6^ A20 cells via the tail vein to generate a murine leukemia model.

### Statistical analysis

Two-tailed Student’s t test was used for statistical comparison between two groups. Wilcoxon rank test was used for the comparison of survival curves. All statistical analysis was performed by using the GraphPad Prism software (version 6.01; GraphPad Software, La Jolla, CA, USA). Values of P < 0.05 were considered statistically significant.

## Results

### HIF-1α inhibitor echinomycin increases Treg development and diminishes alloantigen-specific T helper cell responses ex vivo

**Fig. 1:**
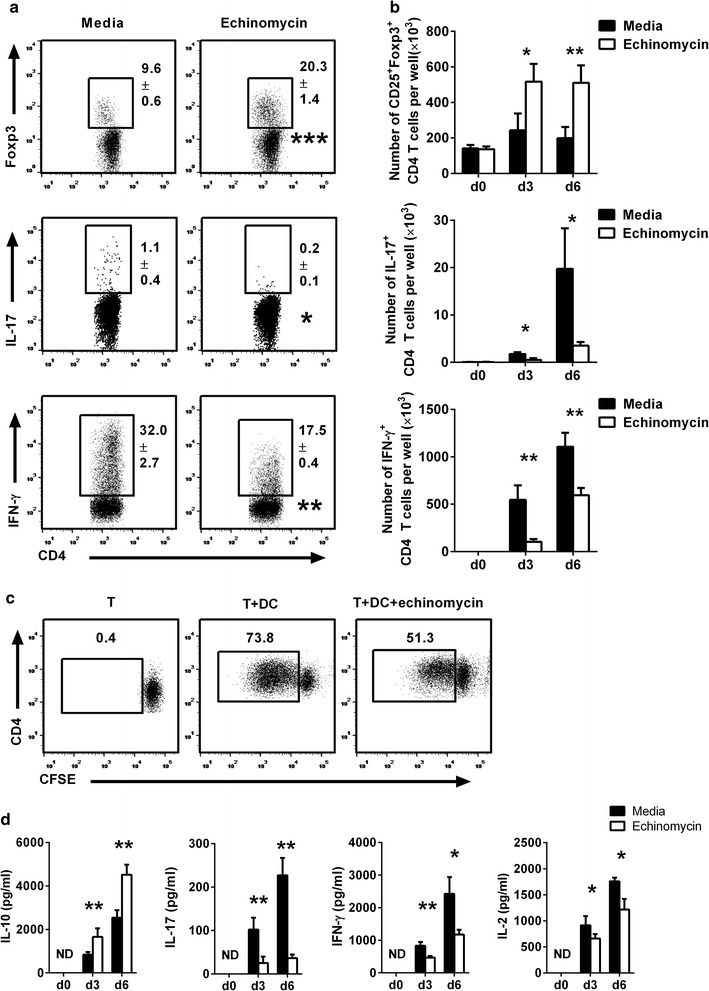
HIF-1α inhibitor echinomycin increases regulatory T cell development during alloantigen-specific CD4 T cell responses ex vivo. Purified splenic CD4 T cells from C57BL/6 mice were cocultured with BMDCs of Balb/c mice. On days 0 (immediately after coculture), 3, and 6 of culture, cells were recovered for intracellular staining and flow cytometry analysis. Representative plots and frequencies of intracellular Foxp3, IL-17, or IFN-γ expression in CD4 T cells on day 6 are shown in **a**. *Numbers* in flow cytometry plots represent mean ± SD frequency (%) of Foxp3^+^, IL-17^+^, or IFN-γ^+^ cells in CD4 T cells. Absolute numbers of Foxp3^+^, IL-17^+^, or IFN-γ^+^ CD4 T cells per well on days 0, 3, and 6 are shown in **b**. **c** Proliferation of CD4 T cells cultured alone (T), with allogeneic DCs (T + DC), or with allogeneic DCs at the presence of echinomycin (T + DC + echinomycin), on day 5 of culture is indicated by CFSE dilution analysis. *Numbers* in flow cytometry plots represent frequency (%) in total CD4 T cells. **d** Concentrations of cytokines in the supernatant on days 0, 3 and 6 of culture are shown. *ND* not detected in either group. *Bar graphs* are shown as mean ± SD. *P < 0.05, **P < 0.01, ***P < 0.001. Data are representatives of three independent experiments with triplicate wells in each group

To determine the impact of HIF-1α inhibition on alloantigen-specific CD4 T cell responses, we cultured BMDCs of Balb/c mice with allogeneic splenic CD4 T cells purified from C57BL/6 mice, in the presence of the HIF-1α inhibitor echinomycin. By using the flow cytometry gating strategy shown in Additional file 1: Figure S1a, frequency of various CD4 T cell subsets including Foxp3^+^, IL-17^+^, and IFN-γ^+^ cells in total CD4 T cells was determined. On day 6 of culture, the average frequency of CD25^+^Foxp3^+^ cells in CD4 T cells in echinomycin treatment group was 20.3%, which was significantly higher than that of 9.6% in control group (Fig. 1a; P < 0.001). Notably, in our experiments Foxp3^+^ cells represented around 80% of CD25^+^ CD4 T cells (Additional file 1: Figure S1b). In contrast to the increased frequency of CD25^+^Foxp3^+^ CD4 T cells, the average frequency of IL-17^+^ CD4 T cells in echinomycin treatment group was 0.2%, which was significantly lower than that of 1.1% in control group (Fig. 1a; P < 0.05). Similar to Th17 responses, the average frequency of IFN-γ^+^ CD4 T cells in echinomycin treatment group was 17.5%, which was significantly lower than that of 32.0% in control group (Fig. 1a; P < 0.01). Kinetic analysis on the absolute number of CD4 T cell subsets on days 0, 3 and 6 showed that the numbers of all the three CD4 T cell subsets were increased after coculture with allogeneic BMDCs (Fig. 1b; P < 0.05 or P < 0.01, as indicated in the figure). In line with the frequencies of CD4 T cell subsets, there were significantly higher number of CD25^+^Foxp3^+^ CD4 T cells but significantly lower number of IL-17^+^ and IFN-γ^+^ CD4 T cells in echinomycin treatment group on days 3 and 6 but not on day 0 immediately after coculture (Fig. 1b; P < 0.05 or P < 0.01, as indicated in the figure). There was no increase in numbers of CD4 T cell subsets on day 3 or day 6 of culture when CD4 T cells were cocultured with syngeneic BMDCs (data not shown). These data suggest that HIF-1α inhibition increases Treg development and reduces Th17 and Th1 responses during alloantigen-specific CD4 T cell responses ex vivo.

We went on to determine the impact of echinomycin on the proliferation of CD4 T cells during alloantigen-specific responses. Flow cytometry analysis on CFSE dilution showed reduced CD4 T cell proliferation in echinomycin treatment group on day 5 of coculture with allogeneic BMDCs (Fig. 1c; Additional file 1: Figure S2a). Further analysis on proliferation data demonstrated that in the two groups there was similar frequency of CD4 T cells went into proliferation (Additional file 1: Figure S2b). By combining intracellular flow cytometry staining and CFSE dilution assay, we observed that in CD4 T cells that went into proliferation, there were significantly higher frequency of Foxp3^+^ CD4 T cells but significantly lower frequencies of IL-17^+^ and IFN-γ^+^ CD4 T cells in echinomycin treatment group as compared with media control group (Additional file 1: Figure S2c). These data further suggest that echinomycin drives alloantigen-specific CD4 T cells into a regulatory T cell-skewed differentiation. As a result, frequency of regulatory T cells in CD4 T cells was increased and Th17 and Th1 responses were inhibited. Consistent to the changes in T cell subsets and proliferation after echinomycin treatment, cytokine analysis on days 0, 3 and 6 of culture showed that there were significantly increased IL-10 production but significantly reduced IL-17, IFN-γ, and IL-2 production in echinomycin treatment group on days 3 and 6 of culture (Fig. 1d; P < 0.05 or P < 0.01, as indicated in the figure). To exclude the possibility that echinomycin may reduce the alloantigen presentation capacity by BMDCs, we determined activation markers including MHC-II, CD86, and CD40 expression in BMDCs cocultured with CD4 T cells in the absence or presence of echinomycin. There were no differences in MHC-II, CD86, or CD40 expression in BMDCs between two groups (Additional file 1: Figure S3). Thus our data suggest that inhibition of HIF-1α by echinomycin reduces the magnitude of alloantigen-specific CD4 T cell responses by increasing the development of Tregs, resulting in reduced CD4 T cell proliferation and development of alloantigen responding CD4 T cells toward Th17 and Th1.

### HIF-1α inhibitor echinomycin reduces aGVHD in mice

**Fig. 2:**
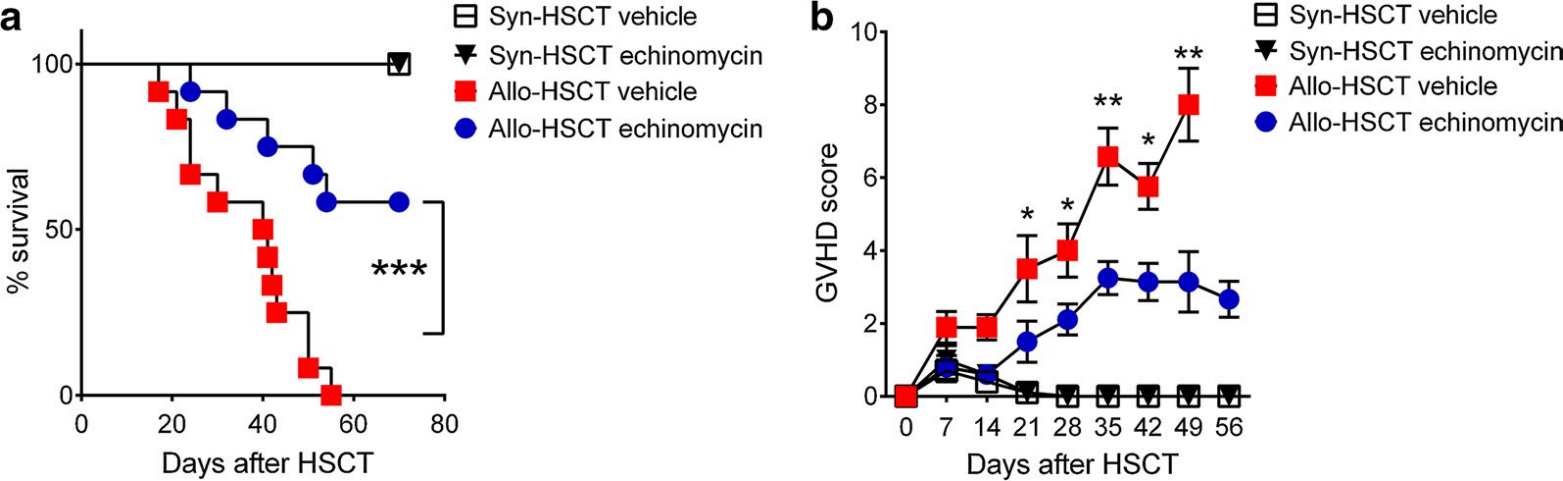
HIF-1α inhibitor echinomycin reduces acute GVHD in mice. Lethally irradiated Balb/c mice were infused with syngeneic or allogeneic bone marrow cells and splenic T cells from Balb/c (syn-HSCT) or C57BL/6(allo-HSCT) mice. Following HSCT, mice were treated with vehicle or echinomycin, and observed daily through day 70 post HSCT for survival (**a**). ***P < 0.001; allo-HSCT vehicle vs allo-HSCT echinomycin. GVHD scores were given weekly based on weight loss, posture, activity, fur texture, and skin integrity (**b**). Data in **b** are shown as mean ± SEM. *P < 0.05, **P < 0.01; allo-HSCT vehicle vs allo-HSCT echinomycin. Data are representatives of two independent experiments with n = 5 in syn-HSCT groups and n = 12 in allo-HSCT groups

We next explored whether this inhibition of alloantigen-specific CD4 T cell responses ex vivo by echinomycin can be translated into reduced aGVHD. By administration of echinomycin following allo-HSCT in a murine aGVHD model, we evaluated the potential impact of echinomycin on aGVHD outcomes. As shown in Fig. 2a, echinomycin treatment significantly prolonged the survival of mice following allo-HSCT (P < 0.001). Consistent with the prolonged survival, GVHD scores were reduced in echinomycin treated mice following allo-HSCT, though the differences were not statistically significant in the first 2 weeks (days 7 and 14; Fig. 2b). And the GVHD scores were significantly reduced in echinomycin treated mice from week 3 through week 7 (days 21–49), as demonstrated by notably reduced body weight loss, hunched posture, ruffled fur, as well as increased activity (Fig. 2b; Additional file 1: Figure S4a–c; P < 0.05 or P < 0.01, as indicated in the figure). These data suggest that HIF-1α inhibition by echinomycin treatment in vivo improves aGVHD outcomes as evidenced by prolonged survival of mice and reduced GVHD scores following allo-HSCT.

### HIF-1α inhibitor echinomycin increases Treg development and diminishes Th17 and Th1 responses during aGVHD

**Fig. 3:**
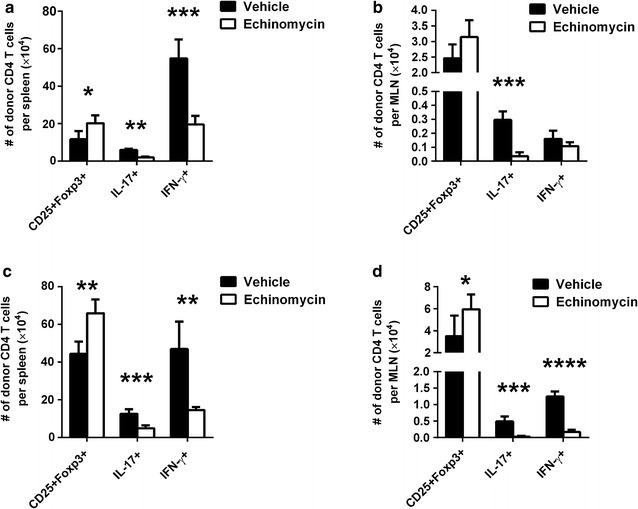
HIF-1α inhibitor echinomycin increases number of Tregs in lymphoid organs in aGVHD mice. Lethally irradiated Balb/c mice were infused with allogeneic bone marrow cells and splenic T cells from C57BL/6 mice to generate aGVHD. Mice were treated with vehicle or echinomycin, and sacrificed on days 14 or 21. Intracellular expression of Foxp3, IL-17, or IFN-γ in donor-derived CD4 T cells in the spleen and MLN were determined. Number of CD25^+^Foxp3^+^, IL-17^+^, or IFN-γ^+^ CD4 T cells on day 14 in the spleen (**a**) and MLN (**b**), as well as on day 21 in the spleen (**c**) and MLN (**d**) are shown. *Bar graphs* are shown as mean ± SEM. *P < 0.05, **P < 0.01, ***P < 0.001, ****P < 0.0001. Data are representatives of three independent experiments with n = 5 per group

To further explore whether the reduced aGVHD in echinomycin treated mice following allo-HSCT was associated with increased Tregs and/or diminished Th17 and Th1 responses as we observed in our ex vivo culture system, we determined the number of the three CD4 T cell subsets on days 14 and 21 in the spleen and mesenteric lymph nodes (MLN) by using flow cytometry analysis. On day 14 following allo-HSCT, there were significantly increased number of CD25^+^Foxp3^+^ CD4 T cells and significantly reduced number of IL-17^+^ and IFN-γ^+^ CD4 T cells in the spleen of echinomycin treated mice (Fig. 3a; P < 0.05, or as indicated in the figure). In MLN, there was significantly reduced number of IL-17^+^ CD4 T cells (P < 0.001) in echinomycin treated mice, though there were no significant differences in the number of CD25^+^Foxp3^+^ or IFN-γ^+^ CD4 T cells (Fig. 3b). On day 21 following allo-HSCT, there were significantly increased number of CD25^+^Foxp3^+^ CD4 T cells and significantly reduced number of IL-17^+^ and IFN-γ^+^ CD4 T cells in both the spleen and MLN of echinomycin treated mice (Fig. 3c, d; P < 0.05, or as indicated in the figure). These data demonstrated that reduced aGVHD by HIF-1α inhibitor treatment in vivo was associated with increased Treg development and diminished Th17 and Th1 responses during aGVHD pathogenesis.

### HIF-1α inhibitor echinomycin preserves GVL effects

**Fig. 4:**
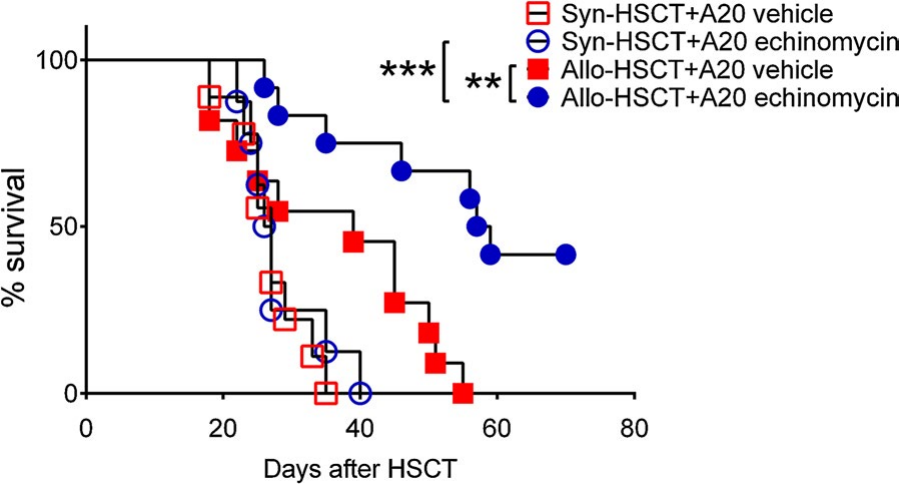
HIF-1α inhibitor echinomycin preserves GVL effect and improved leukemia-free survival. Lethally irradiated Balb/c mice were infused with A20 leukemia cells plus syngeneic or allogeneic bone marrow cells and splenic T cells from Balb/c (syn-HSCT) or C57BL/6 (allo-HSCT) mice. Mice were treated with vehicle or echinomycin, and observed daily through day 70 post HSCT. Survival of animals is shown. ***P < 0.001, allo-HSCT + A20 echinomycin vs syn-HSCT + A20 echinomycin; **P < 0.01, allo-HSCT + A20 echinomycin vs allo-HSCT + A20 vehicle. Data are representatives of two independent experiments with n = 8–11 per group

To evaluate the impact of echinomycin on GVL effects that are critically required for the eradication of residual leukemia cells, we supplemented A20 leukemia cells to the HSC graft, followed by echinomycin treatment as mentioned in our aGVHD model. As shown in Fig. 4, mice receiving syngeneic HSCT (syn-HSCT) and A20 cells died within 40 days post HSCT regardless of whether echinomycin was administered. Survival of mice receiving allo-HSCT + A20 plus echinomycin treatment was significantly prolonged as compared with mice receiving syn-HSC + A20 plus echinomycin treatment (P < 0.001), indicating the presence of GVL effect in echinomycin treated mice following allo-HSCT. Moreover, in mice receiving allo-HSCT + A20 cells, survival was significantly prolonged in echinomycin treated group as compared to that of vehicle group (P < 0.01). These findings suggest that echinomycin treatment preserves GVL effect and thus prolongs leukemia free survival of mice via reducing aGVHD following allo-HSCT.

### Echinomycin does not inhibit A20 growth in vivo

**Fig. 5:**
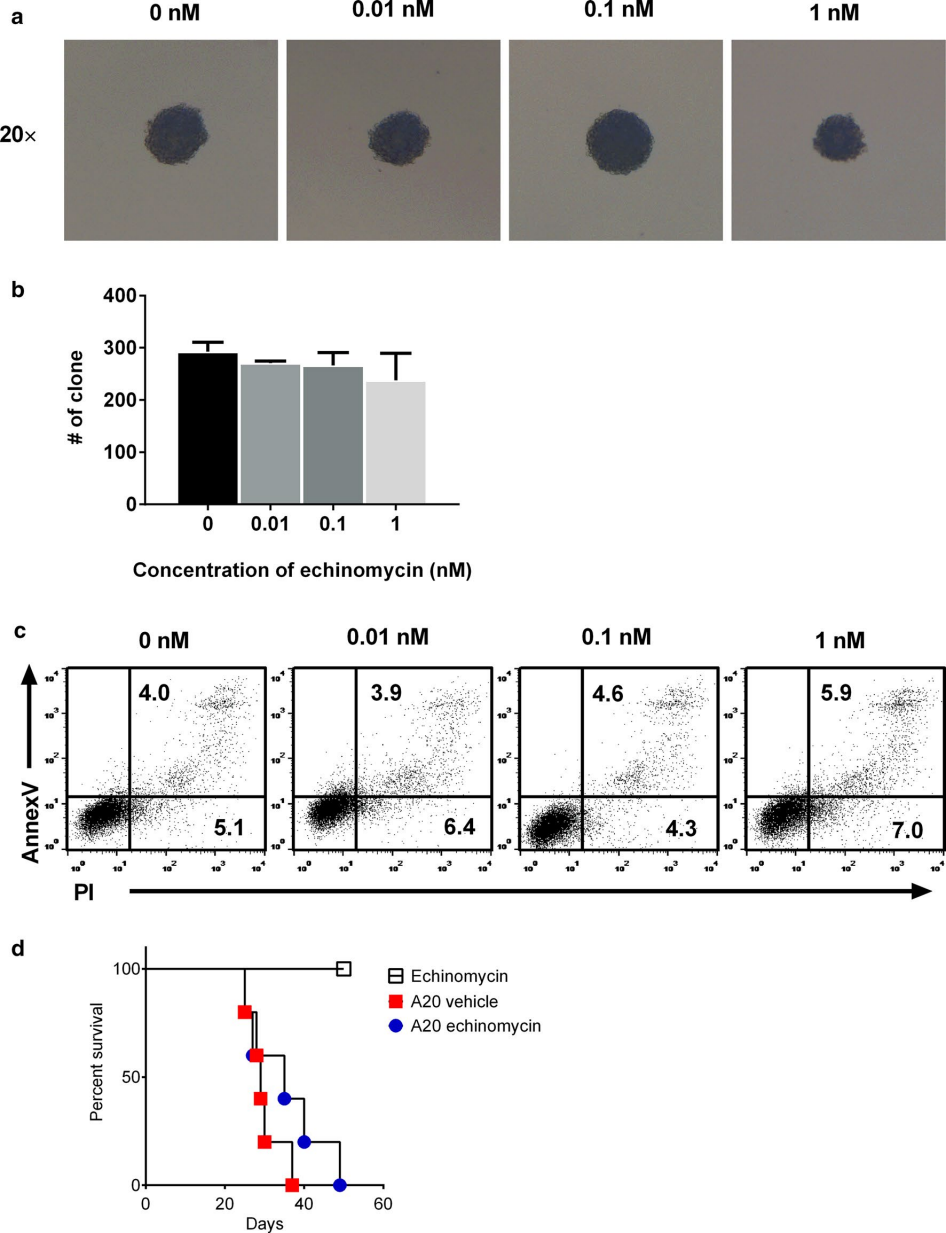
Impact of echinomycin on leukemia cell growth in vitro and in vivo. A20 leukemia cells were pre-treated with various concentrations of echinomycin in vitro, followed by colony forming analysis. Pictures of representative single colony (**a**) and number of colonies (**b**) formed by A20 cells are shown. In **b**, data are shown as mean ± SD. **c** Shows representative flow cytometry dot plots on the apoptosis of A20 leukemia cells treated with various concentrations of echinomycin in vitro. *Numbers* in dot plots represent frequency (%) of cells in the indicated quadrants in total A20 cells. **d** Sub-lethally irradiated Balb/c mice were treated with echinomycin, or infused with A20 cells followed by either vehicle or echinomycin treatment. Survival curves of mice are shown. Data in **a**, **b**, and **c** are representatives of three independent experiments with triplicate wells per group. Data in **d** are representative of two independent experiments with n = 5 per group

HIF-1α inhibition by echinomycin has been shown to inhibit cell proliferation and induce apoptosis in a number of malignant cell lines including myeloid and lymphoid leukemia cell lines [28–31]. Thus the prolonged survival in echinomycin treated mice following allo-HSCT plus A20 infusion might due to direct inhibition of A20 by echinomycin. Although our data in Fig. 4 showed that echinomycin may not have anti-leukemia effect on A20 following a syngeneic HSCT, we used both in vitro and in vivo non-HSCT A20 leukemia models to further address whether echinomycin inhibit A20 cells growth and/or apoptosis in our experimental systems. We cultured A20 cells in vitro in the presence of increasing concentrations of echinomycin from 0 to 1 nM. Representative pictures of colony formation by A20 cells showed that after culture with echinomycin of 0.01 and 0.1 nM there was no notable reduction in the size of single colony (Fig. 5a). However after culture with echinomycin at 1 nM, the size of single colony formed by A20 cells was reduced (Fig. 5a). The numbers of colonies per well were not different across all echinomycin concentrations we used (Fig. 5b). These data suggest echinomycin treatment inhibits A20 cell growth in vitro only at high concentrations. Flow cytometry analysis of Annex V and PI showed that there was no increased apoptosis or cell death in A20 cells after culture in the presence of up to 1 nM echinomycin (Fig. 5c). Importantly, in vivo administration of echinomycin did not prolong the survival of mice infused with A20 (Fig. 5d). These data further demonstrate that in our experimental system echinomycin does not significantly inhibit the growth of A20 leukemia cells in vivo. These findings thus further support our conclusion that treatment with HIF-1α inhibitor echinomycin preserves GVL effect.

## Discussion

Separation of GVHD and GVL has been the focus in allo-HSCT studies for decades [2, 4]. Despite of significant improvements in both basic studies and clinical practice in this field, novel GVHD prophylactic strategies that are superior in efficacy, cost effective, less technically demanding, and without compromising GVL effect are still in urgent need [1, 2]. Here by using murine models of aGVHD and GVL we show that the HIF-1α inhibitor echinomycin reduces aGVHD and preserves GVL effect. The reduction of aGVHD by echinomycin treatment is associated with increased Treg development and diminished alloantigen-specific Th17 and Th1 responses in vivo. As a result, prophylactic echinomycin treatment prolongs leukemia free survival of mice following allo-HSCT.

HIF-1α is a transcription factor that, in CD4 T cells, directly binds to RORγt gene to activate its transcription and cooperates with RORγt and p300 to activate IL-17A gene transcription [18]. As a result, HIF-1α plays a positive role in Th17 differentiation and is required in the long term persistence of human Th17 after adoptive transfer into immunodeficient mice [18, 24]. Concurrently, HIF-1α plays a negative role in Treg development via directly binding to Foxp3 and target Foxp3 protein to degradation. Such a role of HIF-1α in Th17/Treg balance is further evidenced by the findings that mice with HIF-1α deficiency specifically in CD4 T cells were resistant to autoimmune encephalomyelitis (EAE), a Th17 dependent disease [18]. And this EAE resistant phenotype is associated with reduced Th17 responses and increased number of Tregs [18]. Given the contradictory roles of Tregs and Th17 in aGVHD, we hypothesize that pharmacological inhibition of HIF-1α may help to reduce aGVHD via increasing Treg-mediated immune homeostasis and diminishing Th17 responses. To test this hypothesis we started with an ex vivo culture system to determine the impact of the HIF-1α inhibitor echinomycin on alloantigen-specific CD4 T cell responses. When splenic CD4 T cells were cultured with allogeneic BMDCs, there were increased numbers of Tregs on days 3 and 6 as compared with day 0. The increased number of Tregs in the culture indicated the expansion of preexisting splenic natural Tregs and/or the development of alloantigen-specific inducible Tregs. Not unexpectedly, Th17 and to a much larger extent Th1 responses increased over time in the culture. Supplementation of echinomycin, a small molecule inhibitor of HIF-1α, to the culture system resulted in significantly increased development of Tregs and reduced Th17 and Th1 responses. Echinomycin has been shown to reduce the DNA binding activity of HIF-1α, though it remains unknown but possible that echinomycin also reduces protein binding activities of HIF-1α [23]. Based on our data and current information we hypothesize that echinomycin increases Treg development at least partially via reducing the HIF-1α-Foxp3 binding that resulted in reduced Foxp3 degradation. And meanwhile echinomycin diminishes alloantigen-specific Th17 responses via reducing HIF-1α binding to RORγt gene and consequently reducing activation of IL-17A gene transcription. The reduced Th1 responses in echinomycin treatment group may be explained by the increased number of Tregs and therefore augmented regulatory roles of Tregs as a population [21]. In line with such an increased number of Tregs in the culture, there was reduced CD4 T cell proliferation in echinomycin treatment group. These changes in Treg versus T helper cell development are further supported by the respective changes in cytokines IL-2, IL-10, IL-17 and IFN-γ in culture supernatant induced by echinomycin.

Acute GVHD remains a huge challenge to favorable clinical outcomes following allo-HSCT in leukemia patients [2, 4]. It has been clearly shown in both human and mouse that systemic inflammation and target tissue damage during aGVHD attribute largely to donor T cells that are activated upon recognizing the mismatched host major and/or minor HLA (MHC) alleles [1, 4]. Indeed, in both human and mouse, depletion of T cells from HSC graft resulted in reduced incidence and severity of GVHD [9]. Both Th17 and Th1 cells are critically required in the initiation and immunopathology of aGVHD [6, 7, 32]. Tregs, on the contrary, induce immune homeostasis and play regulatory roles in Th1- and Th17-mediated inflammation and tissue damage [21]. It has been shown that the number of Tregs in HSC graft is associated with GVHD [15, 16], and supplementation of donor Tregs either freshly purified or expanded ex vivo to HSC graft resulted in reduced GVHD [13, 14]. Thus, these findings underline the harness of donor Tregs as a promising prophylactic strategy against GVHD. We show here that the HIF-1α inhibitor echinomycin reduces murine aGVHD. And such a phenotype is associated with increased number of donor Tregs in lymphoid tissues. Our study thus provides a novel pharmacological strategy that circumvents the costly, time consuming, and highly technically demanding clinical protocols to enrich and expand Tregs. Compared with Treg infusion, such a pharmacological HIF-1α targeting strategy might be superior in protecting target organ by direct antagonizing Th17 responses.

In addition to GVHD, leukemic relapse remains a major obstacle to favorable clinical outcomes following allo-HSCT [2]. GVL effect is a key to eradicating residue leukemia cells and preventing leukemia relapse [2, 3, 5]. Our data showed that although there were reduced alloantigen-specific CD4 T cell responses in echinomycin treated mice, GVL effect was not weakened. We speculate that there is a threshold level of T cell responses required for GVL effect to eradicate leukemia cells. And in our current study the increased Treg number does not cause T cell responses to be below that threshold. Echinomycin has been shown to inhibit malignant cell growth and induce apoptosis [28–31]. However, we did not observe significant inhibition of A20 leukemia cell growth in vivo by our echinomycin treatment regimen. We speculate that this is at least partially due to that we used a lower dose of echinomycin than previous studies [28, 31]. However, our findings in A20 cells did not exclude the possibility that in certain leukemia cells HIF-1α inhibition remains an effective anti-leukemia regimen. Thus, our data provide compelling evidence that the HIF-1α inhibitor echinomycin preserves GVL effects, presumably via direct targeting HIF-1α. Based on previous data that HIF-1α inhibition by echinomycin preferentially target leukemia-initiating cells without adverse effects on hematopoietic stem cells [28], we further hypothesize that HIF-1α inhibition is a promising prophylactic strategy that not only reduces aGVHD and preserves GVL but also preferentially targets leukemia-initiating cells that helps to further reduce the risk of leukemia relapse.

In conclusion, we have provided evidence that the HIF-1α inhibitor echinomycin reduces aGVHD without weakening GVL effect, which is associated with increased donor Treg development and reduced alloantigen-specific Th17 and Th1 responses in vivo, resulting in significantly prolonged leukemia free survival in mice. Our data thus provide new insights in future studies on the separation of GVHD and GVL via pharmacological inhibition of HIF-1α.

## Conclusions

A small molecule HIF-1α inhibitor echinomycin reduces aGVHD and preserves GVL effect following allo-HSCT in mice. The reduction of aGVHD is associated with increased donor Treg development and diminished alloantigen-specific Th17 and Th1 responses in vivo. Our findings highlight the significance of HIF-1α as a key molecular modulator in aGVHD development. Pharmacological inhibition of HIF-1α thus represents a promising aGVHD prophylactic measure that is worthy of further study.

## Additional file

**Additional file 1.** Additional figures.

## Abbreviations

aGVHD: acute graft-versus-host disease
Allo-HSCT: allogeneic hematopoietic stem cell transplantation
BMDCs: bone marrow derived dendritic cells
CFSE: carboxyfluorescein succinimidyl ester
CFU: colony forming units
DMSO: dimethyl sulfoxide
ELISA: enzyme linked immunosorbent assay
GM-CSF: granulocyte-monocyte colony stimulating factor
GVHD: graft-versus-host disease
GVL: graft-versus-leukemia
HIF-1α: hypoxia-inducible factor 1 alpha
HSC: hematopoietic stem cells
HSCT: hematopoietic stem cell transplantation
IL-2: interleukin-2
IL-4: interleukin-4
IL-10: interleukin-10
IL-17: interleukin-17
IFN-γ: interferon-gamma
LPS: lipopolysaccharide
MLN: mesenteric lymph nodes
PI: propidium iodide
Treg: regulatory T cells
Th17: T helper 17 cells
Th1: T helper 1 cells
