# Supplementary material for: HIF-1α inhibitor echinomycin reduces acute graft-versus-host disease and preserves graft-versus-leukemia effect
Source: J Transl Med. 2017 Feb 10;15:28. doi: 10.1186/s12967-017-1132-9 (PMC5301444; doi:10.1186/s12967-017-1132-9)
Supplement: Supplementary file 1 — Additional file 1. Additional figures. [file 12967_2017_1132_MOESM1_ESM.docx]

**Figure S1. Flow cytometry gating strategy of CD4 T cells and staining of Tregs**

BMDCs of Balb/c were cocultured with purified allogeneic splenic CD4 T cells of C57BL/6 mice. On day 6 of culture, cells were recovered for flow cytometry staining and analysis. (**a**) Representative gating strategy of intracellular staining in CD4 T cells, as presented in Figure 1a. (**b**) Representative plot on Foxp3 expression in CD25^+^ CD4 T cells. Number in the left plot represents frequency (%) of CD25^+^ cells in total CD4 T cells. Number in the right plot represents frequency of Foxp3^+^ cells in CD4^+^CD25^+^ cells. Data are representatives of three independent experiments with triplicate wells.





**Figure S2. Proliferation of CD4 T cells cocultured with allogeneic BMDCs *ex vivo***

Purified splenic CD4 T cells from C57BL/6 mice were labeled with CFSE and cocultured with BMDCs of Balb/c mice in the presence of echinomycin or without echinomycin(media). On days 5 of culture, cells were recovered for extracellular and intracellular flow staining and flow cytometry analysis. (**a**) FlowJo software-based proliferation analysis was performed and generations 0 to 7 of division (G0 to G7) in the two treatment groups were compared. (**b**) Percentage of CD4 T cells that went into proliferation is shown. (**c**) Representative plots and frequencies of intracellular Foxp3, IL-17, or IFN-γ expression in proliferating CD4 T cells are shown. Numbers in dot plots represent mean±SD frequency (%) in total CD4 T cells. Bar graphs are shown as mean±SD. Data in (a) and (b) are representatives of three independent experiments with triplicate wells in each group. Data in (c) are from one experiment with triplicate wells in each group. * p<0.05, ** p<0.01.





**Figure S3. Impact of echinomycin on phenotype of BMDCs cocultured with allogeneic CD4 T cells.**

BMDCs of Balb/c mice were cocultured with purified allogeneic splenic CD4 T cells of C57BL/6 mice in the presence of echinomycin or without echinomycin. On day 3 of culture, cells were recovered for flow cytometry staining and analysis. Representative histograms on MHC-II, CD86 and CD40 expression in BMDCs are shown. Grey filled histograms represent isotype antibody staining control. Data are representatives of two independent experiments with triplicate wells in each group.





**Figure S4. Echinomycin reduces aGVHD in mice.**

Lethally irradiated Balb/c mice were infused with syngeneic or allogeneic bone marrow cells and splenic T cells from Balb/c (syn-HSCT) or C57BL/6(allo-HSCT) mice. Mice were then treated with vehicle or echinomycin. Representative pictures on physical appearance of mice on day 35 following syn-HSCT (**a**; n=5), allo-HSCT treated with vehicle (**b**; n=12) or echinomycin (**c**; n=12) are shown. Data are representatives of two independent experiments.
